# Automated Protein Localization of Blood Brain Barrier Vasculature in Brightfield IHC Images

**DOI:** 10.1371/journal.pone.0148411

**Published:** 2016-02-01

**Authors:** Rajath E. Soans, Diane C. Lim, Brendan T. Keenan, Allan I. Pack, James A. Shackleford

**Affiliations:** 1 Department of Electrical Engineering, Drexel University, Philadelphia, United States of America; 2 Department of Medicine, University of Pennsylvania, Philadelphia, United States of America; 3 Center for Sleep and Circadian Neurobiology, University of Pennsylvania, Philadelphia, United States of America; Hungarian Academy of Sciences, HUNGARY

## Abstract

In this paper, we present an objective method for localization of proteins in blood brain barrier (BBB) vasculature using standard immunohistochemistry (IHC) techniques and bright-field microscopy. Images from the hippocampal region at the BBB are acquired using bright-field microscopy and subjected to our segmentation pipeline which is designed to automatically identify and segment microvessels containing the protein glucose transporter 1 (GLUT1). Gabor filtering and k-means clustering are employed to isolate potential vascular structures within cryosectioned slabs of the hippocampus, which are subsequently subjected to feature extraction followed by classification via decision forest. The false positive rate (FPR) of microvessel classification is characterized using synthetic and non-synthetic IHC image data for image entropies ranging between 3 and 8 bits. The average FPR for synthetic and non-synthetic IHC image data was found to be 5.48% and 5.04%, respectively.

## Introduction

Automatic segmentation of immunohistochemistry images has developed rapidly over several decades, with focus being placed largely on the standardization of clinical pathology. Specifically, great progress has been made in regards to IHC segmentation methods for the diagnoses and staging of cancers using features such as microvessel density and tumor morphology. Such methods, however, have little focus on measuring the concentration of specific proteins within vascular structures. Although critical to the cancer field, features like microvessel density and tumor morphology are less important to neuroscience research.

Measuring the concentration of proteins in microvessels around biological barriers such as the blood brain barrier is vital to our understanding and development of modeling trans-barrier protein delivery. The BBB, in particular, is considered one of the most unique, elusive, and impenetrable barriers in the body and has been studied in conjunction with many diseases such as seizures [1], Alzheimer’s [2, 3] and Parkinson’s [4]. The delivery of treatments across such biological barriers provides a unique and powerful pathway for neuronal degenerative therapeutics—the BBB, in this respect, remains a challenge for targeted pharmaceutical delivery [5–9] and remains an active area of continuing research.

In studying the BBB, IHC is widely used to visualize proteins in a qualitative fashion, giving investigators a valuable tool to survey the distribution of proteins within the brain, specifically its regions (e.g. hippocampus) and subregions (e.g. CA1, CA3, Dentate Gyrus, Perforant Pathway of the hippocampus). A well-recognized concern of IHC is the considerable subjectivity at multiple steps throughout the protocol; thus, preventing IHC from becoming a method for measuring protein concentration. Consequently, Western blots [10, 11] and, more recently, mass spectroscopy [12–14] have been used as a quantitative measure of proteins at the BBB. These techniques measure total protein concentration but lose the spatial distribution of the proteins in the histology. For example, a commonly studied protein in neuroscience is the GLUT1 protein, which is responsible for moving glucose across the BBB. Quantifying the concentration of the GLUT1 protein in a mouse model using Western blots and Liquid chromatography-Mass spectroscopy (LC-MS/MS) requires an average of 10 mouse brains to be combined into one sample in order to provide sufficient protein for analysis. Such methods measure the average GLUT1 concentration of all blood vessels comprising the sample; thereby, losing any information regarding the relative spatial concentration of the GLUT1 protein within the brain’s microvascular structure. Therefore, averaging quantitative methods such as Western blots may only detect a significant change in extreme conditions capable of overcoming the noise floor introduced by the measurement protocol. Consequently, more subtle changes which may be more clinically relevant remain undetected. Although it is possible to quantify GLUT1 proteins at the BBB for a single cryosectioned slice of brain tissue using mass spectroscopy [15–17], this requires considerable resources and is, consequently, widely unavailable.

In this paper, we describe a segmentation workflow that automates the localization of proteins within the microvasculature at the BBB. This method is objective and reproducible, allowing comparative studies between laboratories across various regions and subregions of the brain.

## Materials and Methods

### Animals and Immunohistochemistry

Experiments were approved by the Institutional Animal Care and Use Committees at the University of Pennsylvania in accordance to the National Institutes of Health. We harvested brains of twenty C57/Bl6 male mice, age 4–5 months old (National Institute of Aging, Bethesda). Mice were anesthetized with ketamine, 100 to 200 mg per kg body weight by intraperitoneal injection. After the mouse was under deep anesthesia, we performed an intracardiac perfusion at 120 mmHg, first with Normal Saline for 5 minutes, then with a 4% formaldehyde solution for 5 minutes. Brains were carefully harvested and placed in 4% formaldehyde at 4°C for 24 hours, then cryopreserved with 30% sucrose at 4°C. Brains were then quickly rinsed with tap water, dried and quickly frozen using dry ice. Brains were stored at -80°C until they were sliced on a cryostat in the coronal plane at 40 μm. Slices were stored in a 24 well plate in a 1 to 6 series.

Two mid-hippocampal slices from each of the twenty mice were selected to match Bregma -1.82mm to -2.06mm. All forty slices were stained on the same day by the same laboratory technician using the same solutions. Free-floating slices were first quenched in 60% methanol then blocked before being placed in GLUT1 antibody (rabbit polyclonal at 1:500, abcam, Cat. No. ab652) overnight at room temperature. The next day, slices were put into a biotinylated secondary antibody (JacksonImmunoLab- donkey anti-rabbit) before being amplified with Vectastain Elite ABC (Vector Laboratories). Lastly we incubated the slices in a solution with DAB (final concentration 0.2 mg/mL,Sigma) and Nickel (final concentration 0.3% Sigma-Aldrich) for 20 minutes.

### Image acquisition

Stained hippocampal slices were imaged with a 5–megapixel CCD Bayer Array RGB filter camera (DFC – 425, Leica, Germany) mounted on a digital light microscope (DM5500B, Leica, Germany). Digital images were acquired as 16–bit grayscale, 2592 × 1944 pixel frames and saved in TIFF format with the pixel to pixel distance of 0.5μm at the specimen level. Kohler alignment was performed at the beginning of the microscopy session and again every two hours to ensure standardized conditions. After Kohler alignment at 20× was performed we optimized exposure, gamma, gain and histogram settings for the majority of 40μm cryosectioned slice images stained for GLUT1 expression. Care was taken to prevent non-linearities that could be introduced by saturation and shadowing.

Both the left and right hippocampus were imaged with each side resulting in approximately 9 to 13 z–stacks of images/side with 0–20% overlap between images. On the Leica DM5500B, the top of each z–stack is manually determined as the plane first coming into focus. Due to motorized z–drive feature providing extended depth of field, a 5μm z–stack range was available and selected for all images using a 20× objective with a numerical aperture of 0.7. The z–stack step size was set to 1μm which resulted in the acquisition of six image planes per z–stack by the motorized z–drive system. Thus, a single cryosectioned slice of the hippocampus produced between 18 and 26 z–stacks, or 108 and 156 images planes. Across 20 mice and 40 cryosectioned hippocampal slices, 886 z–stacks were acquired; resulting in a total of 5316 image planes.

### Protein Localization workflow

**Fig 1:**
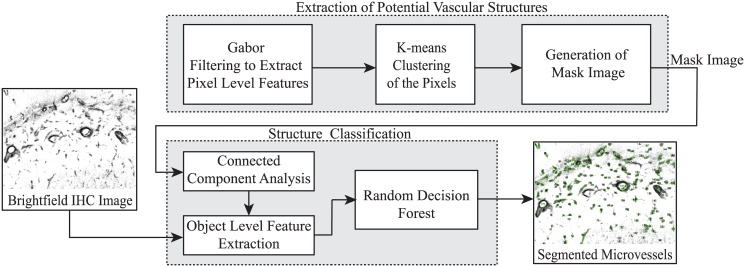
Overview of the workflow. Input immunohistochemistry (IHC) images are pre-segmented to identify candidate structures of interest, which are represented within a generated mask image. Candidate structures within the mask image are filtered using a decision tree derived from training sessions to produce a fully segmented IHC image. For further details, see text.

Blood vessels exhibiting GLUT1 expression are automatically identified, extracted, and parameterized via our custom workflow for performing image segmentation. The workflow combines image based feature extraction techniques with machine learning to form a robust methodology for studying GLUT1 expression with high spatial locality within the brain. The overall workflow is schematically depicted in Fig 1 and consists of two primary stages: (1) a pre-processing stage that isolates vascular structure candidates from the image background, and (2) a feature driven classification stage that identifies true vascular structures among these candidates by using a random decision forest.

### Pre-segmentation of candidate structures

The extraction of relevant vascular structures from IHC images consists of first labeling pixels potentially representing stained vessels. Label map generation begins with identifying pixels that belong to the image regions representing vascular structures. These regions are characterized by their local orientation and spatial frequency, which are extracted using Gabor filtering.

**Fig 2:**
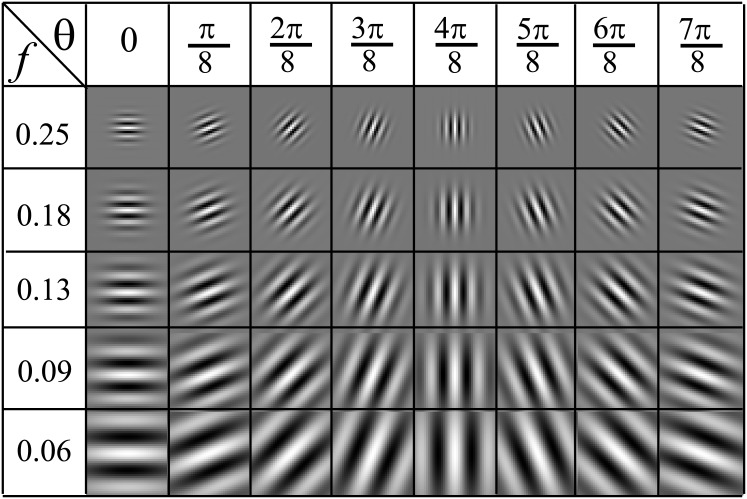
Real parts of the Gabor filter bank. Generated for different combinations of *θ* (in radians) and *f* (in Hz) with $\eta =\sqrt{2}$, $\gamma =\sqrt{2}$ and *ϕ* = 0.

In the spatial domain, a two-dimensional Gabor filter [18] is a Gaussian kernel function modulated by a complex sinusoidal plane wave as defined by Eq 1

$$\begin{array}{r} G(x,y)=\frac{f^{2}}{\pi \gamma \eta }exp\left(-\frac{x^{\prime 2}+\gamma^{2}y^{\prime 2}}{2\sigma^{2}}\right)exp(j2\pi fx^{\prime }+\varphi )\\ x\prime =xcos\theta +ysin\theta \\ y\prime =-xsin\theta +ysin\theta \end{array}$$ (1)

where *f* and *ϕ* define the frequency and phase offset of the sinusoidal factor, respectively. *θ* represents the orientation of the normal with respect to the wave front of the sinusoid. *σ* is the standard deviation of the Gaussian envelope. *γ* and *η* give the spatial aspect ratio specifying the ellipticity of the Gabor function. It is advantageous to generate filters at different scales and orientations since higher frequencies yield finer details and different orientations provides rotation-invariance. A bank of forty Gabor filters varying *f* and *θ* is used to identify frequency and orientation information for each pixel in an IHC image. *f* follows a geometric series with first term equal to 0.25 Hz and a common ratio of $\frac{1}{\sqrt{2}}$. *θ* changes from 0 to 7*π*/8 radians in *π*/8 radian steps (Fig 2).

Pixels potentially representing vascular structures are identified by performing supervised k-means classification on the pixel-wise frequency and orientation information obtained from the application of the Gabor filter bank. The k-means algorithm [19] identifies cluster centers generally referred to as centroids and uses them to initiate clustering. Here, each pixel is assigned to a foreground or background cluster based on the distance between the centroids and the feature values associated with the pixel. The centroids are then recalculated as barycenters of the clusters resulting from the previous iteration. This procedure is iterated until no change occurs. The optimal configuration is achieved by minimizing the total distance between the pixels and the center of the corresponding cluster. Eq 2 shows the objective function using the squared error as the distance metric. The algorithm tries to minimize the squared error function to find the optimal configuration.

$$J=\sum_{j=1}^{k}\sum_{i=1}^{n}\|x_{i}^{\left(j\right)}-c_{j}{\|}^{2}$$ (2)

**Fig 3:**
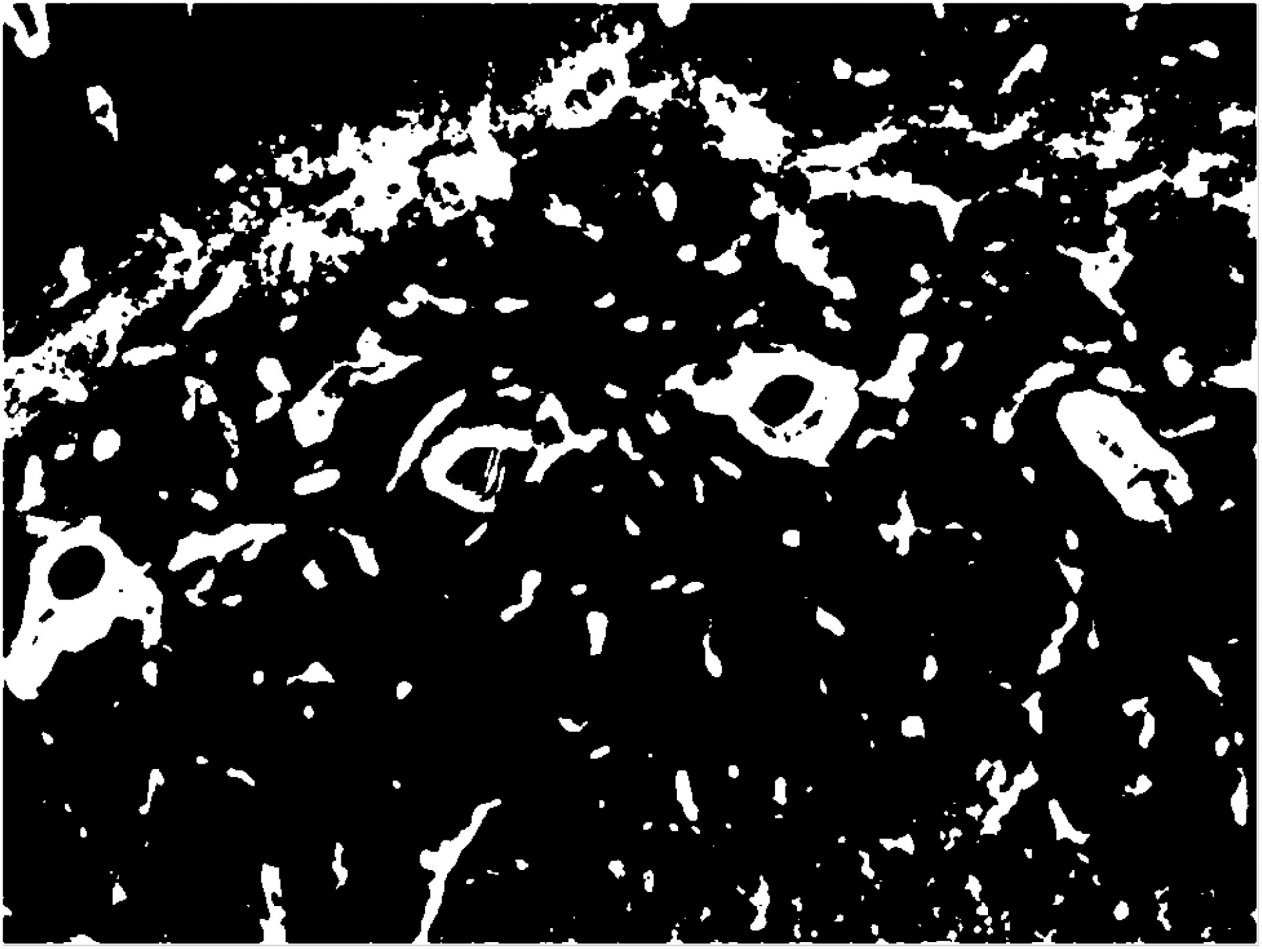
Pixel-wise classification of the hippocampal region using k-means clustering. White pixels mark regions potentially containing vascular structures of interest. Black pixels mark non-vascular structures.

Here, *J* is the squared error, *k* is the number of clusters, *n* is the number of data points, *x_i_* is *i^th^* data point, *c_j_* is the centroid of the *j^th^* cluster and ${\left\|x_{i}^{\left(j\right)}-c_{j}\right\|}^{2}$ is the square of the shortest distance between them. Fig 3 shows a binary mask generated by applying k-means clustering to the features produced by Gabor filtering—white pixels represent areas of the image potentially containing vascular structures relevant to IHC protein localization, whereas black pixels are not of interest. This binary image is used to both mask the IHC image and provide a basis for extracting higher-level structural information about regions of interest by performing connected component analysis on white pixels.

### Connected Component Analysis and Feature Extraction

The identification and extraction of structured pixel information is performed to obtain a higher-level representation of the surface structures. Here, 8-connected neighborhoods are used to identify clusters of pixels corresponding to individual physical structures. The connected component operation [20] assigns temporary labels to each white pixel produced by the k-means clustering algorithm, which are then corrected by an iterative two-pass process that aims to set each pixel’s label to the minimum of its neighbors’ labels. Upon converging, all pixels belonging to a single physical structure are collectively assigned a unique structure label index. This results in a labelmap, which provides the means to iterate through the individual physical structures in the IHC image. All pixels possessing a common label index can then be used to compute various structural features. These features are used to discriminate vasculature containing GLUT1 from physical surface structures not of interest.

Area, diameter, length, solidity, aspect ratio, and intensity features are extracted from each structure produced by the connected-component algorithm. Here, solidity is computed as the ratio of area to convex hull area. The intensity of a structure is obtained by taking the intersection of k-means output with the stained IHC image at the structure of interest and computing the average of the 16-bit grayscale intensity values. Together, these features form the feature vector used for vasculature discrimination, which is performed by an ensemble of decision trees.

### Classification of microvessel candidates using random decision forest

Feature vectors comprised of structural and optical attributes for each structure in the labelmap are used to descriminate between vasculature and irrelevant surface structures. This binary classification is performed using a random decision forest [21] composed of *T* decision trees.

Every tree is grown on an independently drawn bootstrap replica of input data. The predictor variables at each node are identified to classify the data into left or right child nodes based on a binary test, maximizing the information gain.

Generality of the model is enhanced by using an ensemble of trees, known as a random forest. Each decision tree within the ensemble is trained using a random subset of discriminating features from the feature vector in order to avoid overfitting and biased correlation between trees. This allows for the forest to achieve a high probability of classification even for features exhibiting a high variance without introducing additional system bias. When classifying structures obtained from IHC brightfield images, this provides robustness to image noise. Specifically, the lack of correlation between decision trees allows for the ensemble as a whole to remain robust even if an individual tree within the ensemble is highly sensitive to feature noise. Generality is further enhanced by injecting randomness into the model during the forest training phase. At each split node, only a randomly sampled subset *W* of features is considered for training. Node optimization is performed by trying features, *w* ⊂ *W* individually. The tree stops growing when the change in information gain is insignificant.

We consider a structure *x* from the labelmap to be a microvessel if it belongs to class *M*, otherwise the structure belongs to class *N*. For the training procedure, each *x* is associated with a known class label *Y*(*x*) ∈ {*M*, *N*}. At every split node, the binary test *τ*_1_ > *f*(*x*|*w*) > *τ*_2_ is performed and *x* is directed to respective child node. *τ*_1_ and *τ*_2_ are the parameters of the internal split node and *f*(*x*|*w*) is the decision function applied to a structure *x* given the subsampled feature vector *w* ⊂ *W*. The statistical mode of the predictions from individual trees expresses the prediction of the forest as an ensemble.

## Results

### Microvessel Identification in Synthetic Data and Algorithm Validation

Using Gabor filter banks with random forests results in a robust algorithm, which handles varying background complexity. Randomness of the background was modeled using entropy [22] which was defined using 256 bin histograms representing the probability of different grey levels in the image.

**Fig 4:**
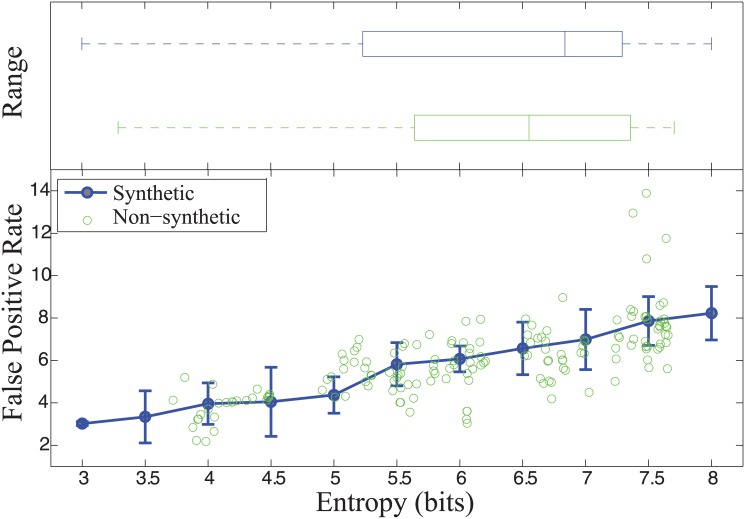
Image Entropy Range and False Positive Rate of microvessel classification. (top) Boxplot showing the median, standard deviation, and range of entropy values for both synthetic and non-synthetic datasets. (bottom) False positive rate (FPR) of microvessel classification for synthetic and non-synthetic IHC images as a function of image entropy.

**Fig 5:**
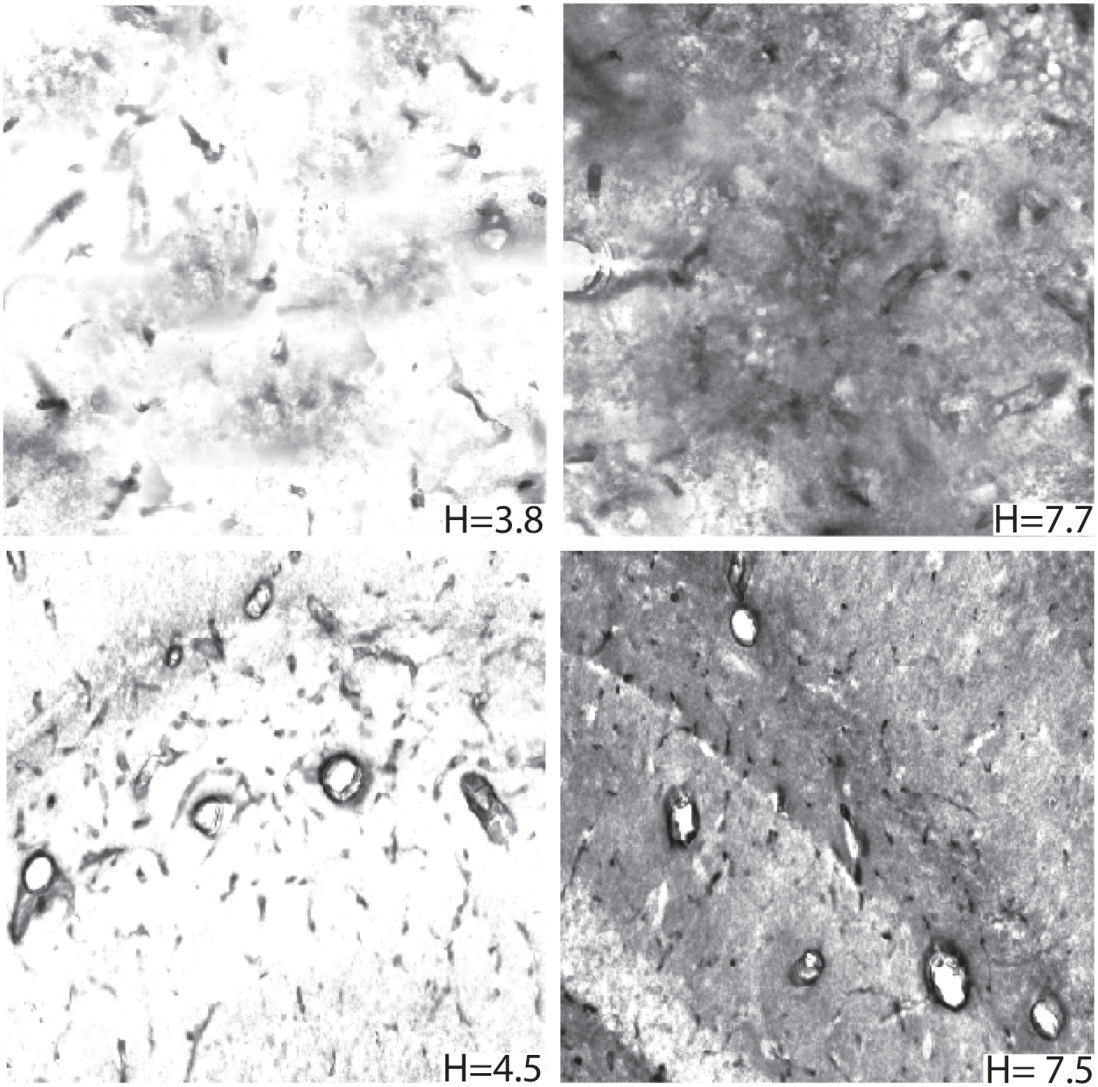
GLUT1 stained image examples. (top) Synthetic and (bottom) non-synthetic images with varying global pixel entropy (*H*) Local spatial frequency tends to increase with local entropy. IHC images with higher *H* usually exhibit more spatially complex surface geometries and/or possess increased surface noise due to the staining protocol.

Synthetic images were generated with known values of entropy and used to test the segmentation algorithm’s sensitivity towards image background complexity. We generated 500 synthetic images using image subregions extracted from 5316 stained GLUT1 images. These subregions were grouped according to entropy. A synthetic image with a specific entropy could then be generated by intelligently piecing together subregions having the necessary entropy while maintaining spatial continuity across subregions. Fig 4 shows the entropy range for synthetic and non-synthetic data. Non-synthetic data had entropy ranging from 3.3 to 7.6 bits and synthetic data had entropy ranging from 3.2 to 7.8 bits. Examples of synthetic images generated are shown in Fig 5.

FPRs were calculated on synthetic images to assess the robustness of the algorithm. Fig 4 shows FPR against entropy values, demonstrating a lower FPR for images with a lower entropy. Specifically, FPR is less than 8% for images with entropy <7; FPR increases up to 14% when entropy approaches 8.

### Microvessel Identification in Non-synthetic GLUT1 Data

**Fig 6:**
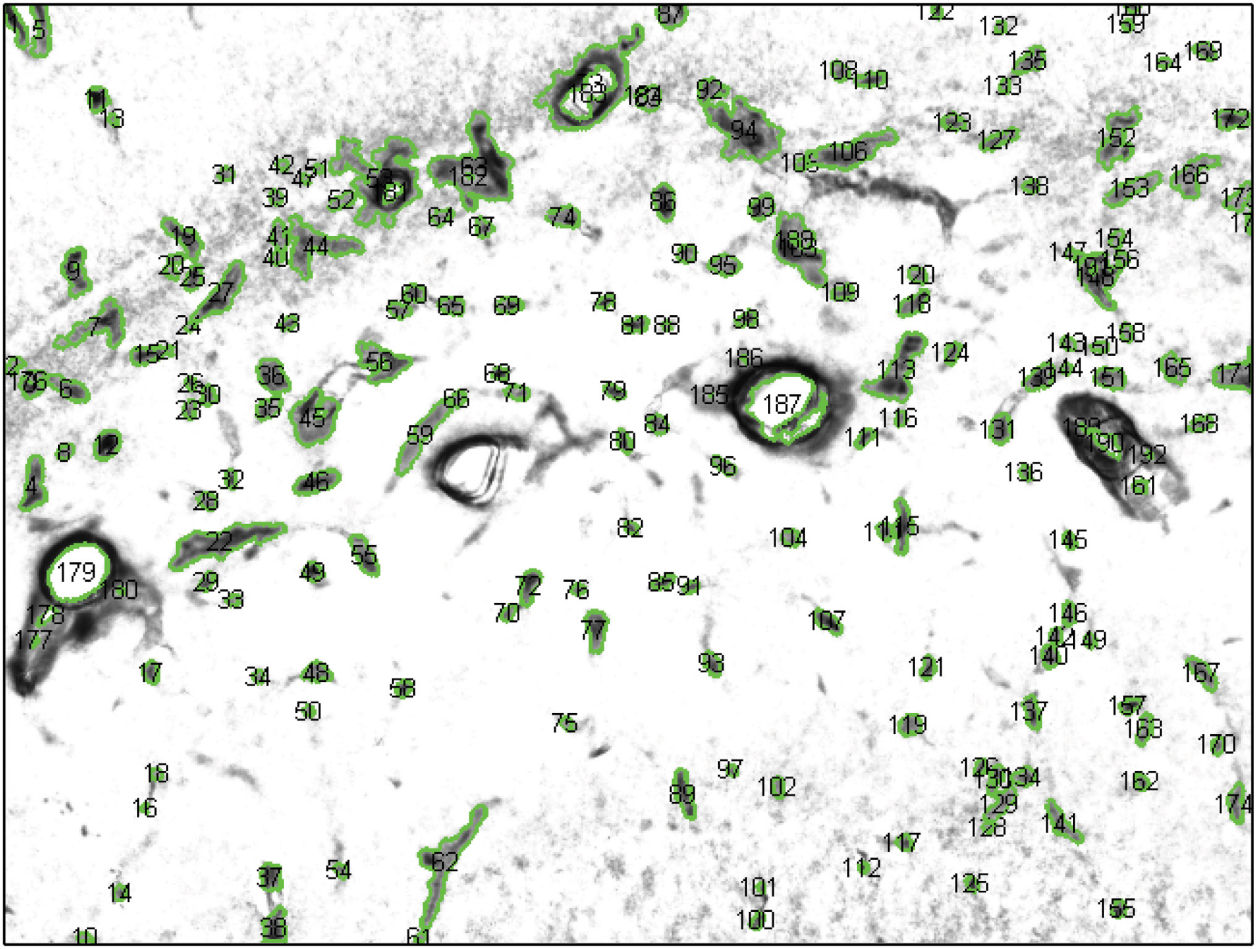
Example result given by the segmentation algorithm. Shown is an IHC image plane within the bregma stained for GLUT1 expression in vascular structures. Green contours identify microvessels exhibiting significant GLUT1 concentration.

The proposed algorithm was employed to perform microvessel identification on 5316 non-synthetic stained hippocampal images (examples shown in Fig 5). Potential microvessel structures present in the label map generated by the pre-processing stage was subjected to decision forest based classification to filter out non-microvessels (arteries, veins and neurons). Fig 6 shows an example of final segmented image. The value of microvessel intensity in the final segmented image quantifies the strength of GLUT1 stain and the variation of intensity over different microvessels provides significant information about relative protein packing in the microvessels.

**Fig 7:**
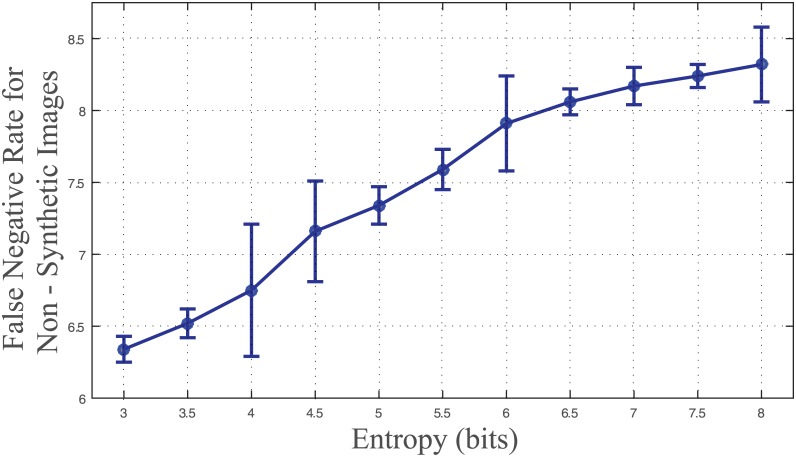
False negative classification rate. FNR of microvessel classification for synthetic data as a function of image entropy. Average FNR = 7.49.

The false positive rate for microvessel identification in non-synthetic images was found to be 5.04% as shown in Fig 4. The false positive rate varies depending upon the location within the hippocampus as it correlates with the degree of image entropy (i.e. background complexity). For example, the perforant pathway is subject to less background complexity and result in false positive rates close to 0. The CA1, CA3 and Dentate Gyrus, however, have more background complexity and consequently a higher false positive rate. Although minimizing false positive rate was the primary goal of the study, an analysis of false negative rate was also performed. Fig 7 shows the variation of false negative rate as a function of entropy.

### Timing and Performance

**Fig 8:**
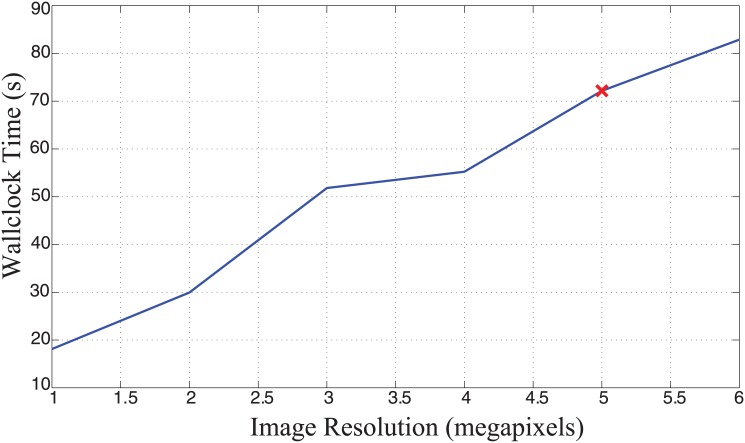
Processing time. The red line marks 5 megapixels, which correlates to the 2592×1944 brightfield IHC images acquired.

Table 1 shows the time required for each stage in the workflow for processing images through a single-threaded CPU implementation using MATLAB. Fig 8 shows the processing time per megapixel of the image.

**Table 1:** Execution time profile for the automated protein localization algorithm.

Operation	Wallclock Time (s)
Gabor filtering	41.99
K-means clustering	24.09
Connected component analysis and feature extraction	1.37
Classification (random forest)	4.70
Total	72.16

Time profile reported here is for performing vasculature segmentation on an 5MP image

The tests reported in this section were performed on a machine with Intel Core i7-3770K processor clocked at 3.50GHz and 32 GB of RAM and analyzed for 10 capillaries per megapixel of the image. Each capillary would contribute an additional 0.29s to the total time required for processing the image.

## Discussion

In this paper we have developed and characterized an objective method for automatic protein localization within hippocampal microvessels at the BBB in stained brightfield histology z-stack images. Characterization of this method using synthetic and non-synthetic images with entropies varying from 3 to 8 bits shows that microvessles expressing GLUT1 concentration can be identified and spatially localized with a worst-case FPR of approximately 9%, where the average FPR is shown to increase monotonically with image entropy. Across 5316 non-synthetic brightfield images of hippocampal subregions stained for GLUT1 expression, the average entropy was found to be 6.6 bits with a standard deviation of approximately 1 bit, which results in a non-synthetic “real-world” image FPR centered around 6%.

The protein localization capabilities afforded by our method provide a means to study the relative distribution of a specific protein across various subregions of the hippocampus, which may enable subsequent research to expand our understanding of protein function with respect to location within the brain. Furthermore, with careful calibration, our method can be extended by exploiting the Beer-Lambert relation [23] between absorbance and solute concentration for a given protein stain. Presently, the value of intensity simply indicates the relative strength of the GLUT1 stain, and the variation in intensity over different microvessels provides significant information about relative protein packing in the microvessels. By integrating the relation between stain intensity and concentration afforded by the Beer-Lambert Law, it would become possible to measure a specific protein’s concentration with spatial locality across the hippocampus. This would provide a powerful tool for furthering the field of drug delivery and our understanding of BBB penetrability.

**Fig 9:**
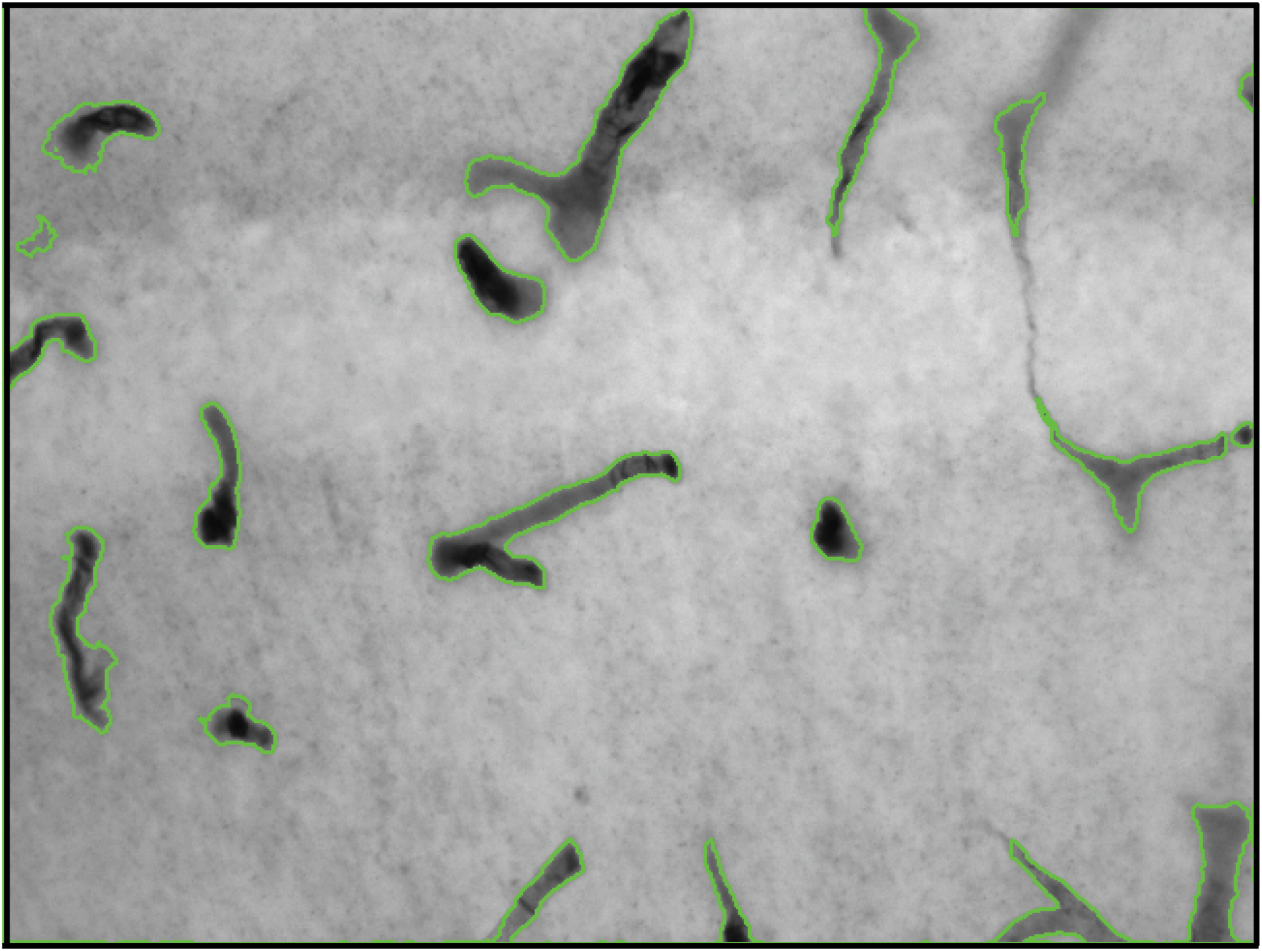
Example result given by the segmentation algorithm when a 40× image is input. Green contours identify microvessels exhibiting significant stain concentration.

In contrast with existing fluorescence based microscopy methods, our proposed localization method does not suffer from the rapid and varied signal decay exhibited by fluorescent stains when exposed to brightfield and does not require the expense of a slide scanner to obtain reliable measurements. Consequently, our brightfield based method does not suffer from magnification restrictions often associated with slide scanners and can be used to analyze images obtained with any arbitrary microscope objective. Fig 9 shows a segmented brightfield z-stack image slice obtained at 40×. By using z-stack images we eliminate focus bias, and by imaging an entire region we eliminate selection bias; thereby, providing a robust methodology.

With further development, our algorithm will be made open source. We will institute a user-friendly interface to allow investigators who are not computer scientists to be the end user. Other future directions include: (1) adding features such as a “magnetic lasso” to draw a region of interest to minimize overlap when taking images on the microscope; (2) algorithm optimization to acquire images at a lower magnification to decrease the time for image acquisition; and (3) providing quantitative expression of proteins in reconstructed 3D vessels. By making this open source, we encourage other investigators to develop the algorithm to identify vessels in other organs and cells such as neurons, microglia, etc.

## Conclusion

Our algorithm provides an automated protein localization method that preserves the spatial distribution along the microvessels at the blood-brain barrier. In our pre-segmentation stage, k-means clustering is employed to identify the pixels forming vascular structures using information extracted by a gabor filter bank. Surface structures are then constructed by performing connected component analysis on identified pixel clusters, and then microvessels are identified from these surface structures using a trained random decision forest. Our proposed imaging protocol results in an objective method as it eliminates both selection and focus bias. Therefore, using widely accessible equipment found within most research facilities, our method enables comparative studies on relative distribution of proteins calculated between experimental conditions with similar or different interventions.
